# Lifestyle Habits Related to Internet Use in Adolescents: Relationships between Wellness, Happiness, and Mental Health

**DOI:** 10.3390/children11060726

**Published:** 2024-06-14

**Authors:** Sigita Lesinskienė, Rokas Šambaras, Agnė Butvilaitė, Justina Andruškevič, Margarita Kubilevičiūtė, Urtė Stanelytė, Severija Skabeikaitė, Izabelė Jūraitytė, Ieva Ridzvanavičiūtė, Kamilė Pociūtė, Natalja Istomina

**Affiliations:** 1 Clinic of Psychiatry, Institute of Clinical Medicine, Faculty of Medicine, Vilnius University, 01513 Vilnius, Lithuania; rokas.sambaras@mf.vu.lt (R.Š.); kamile.pociute@mf.vu.lt (K.P.); 2 Faculty of Medicine, Vilnius University, 01513 Vilnius, Lithuania; agne.butvilaite@mf.stud.vu.lt (A.B.); justina.andruskevic@mf.stud.vu.lt (J.A.); margarita.kubileviciute@mf.stud.vu.lt (M.K.); urte.stanelyte@mf.stud.vu.lt (U.S.); severija.skabeikaite@mf.stud.vu.lt (S.S.); izabele.juraityte@mf.stud.vu.lt (I.J.); ieva.ridzvanaviciute@mf.stud.vu.lt (I.R.); 3 Institute of Health Sciences, Faculty of Medicine, Vilnius University, 01513 Vilnius, Lithuania; natalja.istomina@mf.vu.lt

**Keywords:** problematic internet use, internet addiction, adolescent, mental health, internalizing difficulties, externalizing difficulties, wellness, happiness

## Abstract

Background: Studies in recent years have shown a significant increase in problematic internet use. Prolonged internet usage negatively impacts various aspects of adolescents’ lifestyles. Methods: The survey was conducted in three Lithuanian cities—Vilnius, Klaipėda, and Molėtai—between October and December of 2023. The survey involved students between the ages of 12 and 16. The participants completed a questionnaire that included questions about wellness and happiness, the Internet Addiction Test (IAT), and the Strengths and Difficulties Questionnaire (SDQ). Our study aimed to investigate problematic internet use among Lithuanian adolescents and its relationships with wellness, happiness, and mental health. Results: This study included 1412 adolescents, with a mean age of 14.41 ± 1.20, with the sample composed of 52.3% boys. Boys indicated that they felt healthier and happier than girls (69.4% vs. 47.4%; 51.4% vs. 29.6%), *p* < 0.001. A total of 20.8% of students had IAT scores that reflect a normal level of internet usage. For IAT, the average score of girls was statistically significantly higher than that of boys (41.87 ± 12.24 vs. 39.96 ± 12.37), *p* < 0.001. The IAT score was higher in the group of students who felt less happy and healthy, had more frequent headaches or abdominal pain, and had problems with sleep, *p* < 0.001. A statistically significant, positive weak correlation was found between students’ IAT scores and SDQ internalizing and SDQ externalizing scores (r = 0.366; r = 0.385, *p* < 0.001). Conclusions: Prolonged internet use is arising as a serious problem among adolescents. Negative feelings, unhappiness, and poor emotion regulation have an association with internet addiction. Also, the female gender may be more associated with possible internet addiction. The results of this study can serve as initial steps toward developing preventive interventions.

## 1. Introduction

Over the past decade, it has become nearly impossible to imagine the younger generations’ lives without the internet. From their very first steps, the internet has become an integral part of children’s daily lives. Its pervasive influence is undeniable, shaping their habits and experiences in ways that will last a lifetime. With the increased accessibility to the internet and more unstructured time in daily life, there is a higher risk of overuse, which can result in severe consequences. Most countries face this problem, and ways to prevent excessive internet use, especially when faced with these problems by young people, are constantly being sought. Epidemiological studies have found varying rates of problematic internet use across different countries, ranging from 0.8% in Italy to 26.7% in Hong Kong [1], with global prevalence estimates of 6% [2]. The differences in prevalence rates can be attributed to variations in assessment tools, cutoffs, and participant demographics across surveys. Socio-cultural differences are also cited as a contributing factor by some researchers [3]. Over the past decade, the almost global widespread use of smartphones has also led to many adolescents and children dedicating a substantial amount of their time to digital media consumption [4]. The quick worldwide spread of the internet has prompted numerous studies revealing issues linked to the growing prevalence and regularity of internet usage. This includes a pervasive problem known as internet addiction, recognized as a new form of addiction arising from excessive online engagement [5].

The term “Internet-related disorders” refers to addictive patterns of internet use, which are now categorized as behavioral disorders. Individuals experiencing these issues often display dependence, typically on specific internet activities like computer games, social networks, and online pornography. Such behaviors increasingly align with the addiction criteria outlined by the World Health Organization (WHO) [6]. Internet addiction is defined by an excessive or inadequately managed fixation, impulses, or actions related to computer usage and online activities, resulting in functional impairment or emotional distress [7]. It is characterized by a diminished sense of the value of time when not using the internet, heightened irritability and aggression when denied access to the internet despite the desire for it, and the disturbance of an individual’s social, professional, and familial aspects of life [5]. Addiction criteria also include strong desires, an inability to regulate the start and stop of internet use, the experience of withdrawal symptoms when not online, the need for more internet time to achieve the same satisfaction, ignoring of other responsibilities and hobbies, and persistence with internet use despite adverse effects [6].

Prolonged internet usage negatively impacts various aspects of adolescents’ lifestyles. It can lead to erratic eating patterns, a sedentary lifestyle, reduced sleep duration, and heightened tobacco consumption [8]. Also, excessive use of the internet can cause problems related to a young person’s physical health, such as over- or under-weight, fast food consumption, migraines, and back pain [9]. Furthermore, extensive research has found that internet addiction could lead to bad outcomes, such as poor sleep and a higher risk of developing high blood pressure and low high-density lipoprotein (HDL) cholesterol [2,10]. It could even reduce bone density and lead to osteoporosis [11]. Children and adolescents are very vulnerable; bad outcomes affect them significantly. There is also a psychiatric comorbidity between internet addiction and mood, anxiety, impulse control, and substance use disorders, increasing gambling risk [7,12]. Further, more severe clinical conditions are also associated with internet addiction, such as dysthymic, bipolar, affective, and social anxiety disorders, as well as major depression [13].

Moreover, problematic internet use is associated with several difficulties that arise in school. Demir and colleagues found that internet addiction decreased academic motivation and increased academic procrastination, showing that addicted adolescents are more likely to prioritize their time on the internet, leaving their schoolwork unattended [14]. It can also be noted that problematic internet use may factor in social withdrawal, including school refusal behaviors [15,16]. Also, unsatisfactory school climate and poor relationships with peers and teachers were linked to increased internet use, particularly in older students [17]. This may indicate that a positive school atmosphere protects against problematic internet use.

Scientists unanimously agree that increased internet use has evident links to the COVID-19 pandemic. Due to its high contagiousness and rapid spread, the SARS-CoV-2 virus soon became an immense hazard. A global outbreak of coronavirus disease led 188 countries worldwide to close schools [18]. Digital distance learning became the only way to control infection transmission and pursue education safely. Consequently, even though online learning and virtual communication have brought various advantages, internet usage during quarantine has become more frequent, time-consuming, and addictive, which has increased the risk of compulsive internet use. Screens became frequently used during the quarantine, because they help to relieve stress and overcome boredom and anxiety. One of the coping methods during the coronavirus disease outbreak was gaming, but despite all the positive impact, it escalated gaming disorder prevalence. Furthermore, the lack of face-to-face interaction increased the frequency of social media use. Indeed, it helped children and adolescents worldwide to keep in touch with relatives, friends, and peers [19,20]. Nevertheless, the excess of virtual socialization resulted in 43.69% of adolescents in Germany showing signs of problematic internet use, such as negative emotions while not using the internet, inability to stop using the internet, using the internet to improve bad mood, feeling dominated by the internet, and deterioration of interpersonal relationships. For comparison, the prevalence rate of problematic internet use in different countries before the quarantine was approximately 25% [21]. Another meta-analysis study showed that ~13.6% of teenagers faced the social problem of addiction before, and 24.4% after the coronavirus lockdown [22].

The problem of decreased socialization during the COVID-19 pandemic also resulted in a greater fear of missing out (FOMO). This personality construct is defined as the constant need to possess up-to-date information in apprehension that something important will be missed. Excessive internet use became the primary way to relieve FOMO-associated stress and fulfill social needs. Consequently, the fear of missing out might be another factor that induced problematic smartphone use, more frequent gaming disorder, and internet use in general during the pandemic [20].

Most studies that investigate adolescent internet addiction focus on comorbidity with psychiatric disorders. This clearly indicates that this relationship is closely related to anxiety, attention deficit hyperactivity disorder (ADHD) symptoms, depressive disorders, social phobia, behavioral problems, and hostility, particularly among male adolescents [23,24]. However, much less research focuses on subjective feelings of happiness or well-being. Most of the research in this area is about young adults and university students. Research indicates that adolescents may seek pleasure and escape problems by spending time on the internet [25]. In an attempt to enhance the enjoyable experience, adolescents often form addictive habits [26]. It is suggested that adolescents who seek pleasure in life are more likely to engage in internet-addictive behavior. However, though engaging in the online world may provide temporary pleasure, it may not contribute to their long-term well-being. Research suggests that engagement with the internet does not promote personal development or facilitate the establishment of genuine social relationships [27]. Several studies suggest that poor self-rated health, subjective unhappiness, and depressive symptoms are significantly associated with internet addiction in both boys and girls [28,29]. Nevertheless, there is still a lack of research in this area. Our research focuses on middle adolescence to determine adolescents’ addiction to the internet and their subjective feelings of happiness and health. In this way, the aim is to widen the knowledge about the possible associations between the use of the internet and the feelings of happiness and health of middle-aged adolescents. This research is exclusively new in the Baltic States, as it is noticeable that these studies are significantly lacking in this region.

The excessive use of the internet poses a significant challenge for our society, and it is especially concerning for young people. We must take action to address this issue, as it can have serious long-term consequences if left unchecked. Parents, teachers, and medical professionals should pay attention and notice signs of problematic internet use and addiction.

Our study aimed to investigate problematic internet use among Lithuanian adolescents and its relationships with wellness, happiness, and mental health. We assumed that a more significant addiction to the internet would be in the adolescent group who had more significant psychoemotional difficulties. In this study, we hypothesized that a more significant internet addiction would be in the adolescent group who were less happy and also reported feeling less healthy. We also hypothesized that adolescents who had higher internet addiction scores were more likely to experience disturbed sleep and more frequent abdominal pain or headaches. We further considered that the harmful use of the internet, psychoemotional difficulties, and the feeling of happiness could differ between adolescent girls and boys.

## 2. Materials and Methods

### 2.1. Participantsand Procedure

This survey was organized on an initiative and voluntary basis in close cooperation with the Vilnius University Faculty of Medicine and the Ministry of Education, Science and Sport. The three cities in Lithuania were selected for the study. One of them was Vilnius, which is the capital of Lithuania. According to the Lithuanian Statistics Department, in 2024, Vilnius has a population of 633,917, and there are 76,686 children from 7 to 17 years old. Klaipeda is the largest city in western Lithuania. According to the Statistics Department of Lithuania, in 2024, Klaipeda has a population of 172,031, and there are 20,870 children between the ages of 7 and 17. The city of Molėtai is in the eastern part of Lithuania. In 2024, Molėtai district has a population of 17,712, with 1487 children from 7 to 17 years old. In each city, we interviewed students from one secondary school and one high school. Six schools (3 secondary and 3 high schools) in three Lithuanian cities, Vilnius, Klaipeda, and Molėtai, were invited, and all agreed to participate in the anonymous survey. The research conducted in schools had full authorization from the Ministry of Education, Science, and Sport. The researchers contacted school administrations, who agreed to conduct the study in the selected schools. Later, the school administration informed the student’s parents about the ongoing investigation and obtained parental permissions. Secondary school students in grades 7 and 8 and high school students in grades 9 and 10 were interviewed through an anonymous questionnaire. The survey was conducted in schools between October and December of 2023. Participants were informed about the aims and scope of the survey. All participants filled out the paper questionnaires of their own free will. Researchers interviewed those teenagers whose parents gave permission to participate. After the informed consent, adolescents voluntarily filled in the questionnaires. The study aims and purposes were described to the adolescents, and they were invited to join the research project anonymously and voluntarily. Following this introductory part, all students agreed to complete the questionnaire. Some of the respondents did not provide answers to specific questions, but overall, the response rate was acceptable. We observed active involvement and interest in the research subject among the participants.

### 2.2. Questionnaire

The authors developed the original anonymous questionnaire for research purposes. It consisted of five sections, designed and titled to provide more clarity and visual structure for the respondents.

The questions were divided into separate sections and comprised socio-demographic data (age; gender female/male; place of school: Vilnius/Klaipeda/Molėtai; grade: 7/8/9/10; family status: living with both parents/living with one parent), a section on aches and sleep (during the past six months, have you experienced distracting headaches? Hardly ever/Less than once a month/At least once a month/At least once a week. During the past six months, have you experienced recurring abdominal pain? Hardly ever/Less than once a month/At least once a month/At least once a week. During the past six months, have you experienced problems with falling asleep or sleeping? Never/<1 time a week/1–2 times a week/3–5 times a week/every night), questions about students’ well-being (Are you feeling healthy? Yes/Partially/No; Are you feeling happy? Yes/Partially/No).

Emotional and behavioral problems were measured by the self-reported version of the Strengths and Difficulties Questionnaire (SDQ). The SDQ is a commonly employed tool for behavioral screening, providing a fast and user-friendly way to measure both mental health challenges and strengths. Initially designed, it aimed to measure five facets of mental health screening: four domains focused on difficulties (hyperactivity/inattention, emotional, conduct, and peer) and one domain centered on strengths (prosocial behavior) [30]. Our study used the SDQ questionnaire, which was validated in Lithuania [31]. The SDQ questionnaire is composed of 25 Likert-type items divided into five scales. The first four scales measure emotional symptoms, behavioral problems, hyperactivity, and peer relationship problems, and the fifth scale measures pro-social behaviors. The total difficulties score (TDS) is generated by adding scores from all the scales except the prosocial scale. The resultant score ranges from 0 to 40. For our study, we also used the internalizing and externalizing score subscales: the internalizing score (IS) (range 0–20) is the sum of the emotional and peer problems; the externalizing score (ES) (range 0–20) is the sum of the conduct and hyperactivity scales. Higher estimates, both in the total scale and in the externalized and internalized scales, indicated greater emotional and behavioral difficulties. The internal consistency coefficient for the total difficulties score was acceptable (0.74). Cronbach α values for emotional symptoms, peer problems, hyperactivity, and the conduct problem scale were 0.74, 0.72, 0.70, and 0.71, respectively. The lowest Cronbach α was found on the prosocial behavior scale (0.63).

The Internet Addiction Test (IAT) measures problematic internet use (internet addiction) [32]. The Internet Addiction Test (IAT) is widely used in Lithuania [33]. The IAT total score is the sum of the ratings given by the examinee for the 20-item responses. Each item is rated on a 5-point scale ranging from 0 to 5 (e.g., How often do you stay online longer than you planned? 0 = never; 1 = seldom; 2 = occasionally; 3 = frequently; 4 = very often, 5 = always). The maximum score is 100 points. The higher the score, the higher the severity of the problem. Total scores that range from 0 to 30 points are considered to reflect a normal level of internet usage; scores of 31 to 49 indicate the presence of a mild level of internet addiction; 50 to 79 reflect the presence of a moderate level; and scores of 80 to 100 indicate a severe dependence upon the internet. The internal consistency coefficient (Cronbach alpha) on this sample was good (0.82).

### 2.3. Statistical Analysis

The statistical analysis (comparisons) was performed according to the background variables. The main background variables included gender, grade, and place of school. Additional variables included headaches, abdominal pain, problems with falling asleep or sleeping, feelings of happiness, and feelings of health. Background variables were compared with such numerical variables as the total difficulties score of the SDQ, the internalizing score of the SDQ, the externalizing score of the SDQ, and the Internet Addiction Test. The normality of the variables’ distribution was tested using the Shapiro–Wilk test. Numerical variables distributed according to the normal distribution were presented with mean and standard deviation. Pearson’s chi-squared test (χ^2^) was used to determine whether there was a statistically significant difference between the expected frequencies and the observed frequencies in one or more categories. Fisher’s exact test was used instead if more than 20% of predicted values were lower than 5. Differences between background variables were investigated using one-way analyses of variance (ANOVA) with more than two independent groups. Comparisons between groups were performed using the post hoc Tukey test. Pearson’s correlation was used to determine the correlation between numerical variables. Additionally, linear regression analysis was performed to control for the variables during assessing the association between the Internet Addiction Test and total difficulties score of the SDQ, the internalizing score of the SDQ, the externalizing score of the SDQ, and gender and age. The indicators of a suitable linear regression model, according to which reasonable conclusions can be drawn, included the following: determination coefficient R^2^ > 0.20; ANOVA *p* < 0.05; Cook’s distance < 1. Differences were considered statistically significant when *p* < 0.05. The data were analyzed by IBM SPSS Statistics (version 22) and Microsoft Excel 2016.

## 3. Results

### 3.1. Main Characteristics of the Sample

This study included 1412 adolescents, with a mean age of 14.41 ± 1.20 (12–16 years old). Males and females were similarly represented in the sample: 738 (52.3%) males and 674 (47.7%) females. Almost half (673 (47.3%)) of the interviewed students were from Vilnius. The distribution of students by class was relatively similar and varied from 22.7% to 26.8%. Four-fifths or 1186 (83.7%) adolescents lived together with two parents. The whole characteristics of the sample are presented in Table 1.

### 3.2. Students’ Aches, Sleep, and Wellness

In the last 6 months, 418 (29.8%) surveyed students, or almost a third, had no headache. However, 317 (22.6%), more than a fifth, experienced distracting headaches at least once a week. Also, over the last 6 months, 435 (31.0%) students did not experience abdominal pain. Meanwhile, 202 (14.4%) students experienced abdominal pain at least once a week. A total of 394 (28.0%) respondents indicated no problems with falling asleep or staying asleep during the 6 months. And 172 (12.2%) students mentioned having problems with falling asleep or sleeping every night. A total of 811 (58.9%) students, i.e., more than half, felt healthy, and 562 (40.9%) felt happy.

Comparing the frequency of headaches in the last 6 months between the genders, it was observed that girls reported having a headache at least once a week, much more often than boys (33.3% vs. 12.8%), *p* < 0.001. Girls also mentioned much more often than boys that they felt abdominal pain at least once a week (22.9% vs. 6.7%), *p* < 0.001. However, there was no significant difference in the occurrence of headaches and abdominal pain between 7th–8th grade and 9th–10th grade students and between students who live with both parents and those who live with one parent. In addition, it was observed that girls indicated twice as often as boys that they had trouble falling asleep or sleeping every night in the last 6 months (16.4% vs. 8.4%), *p* < 0.001. However, there was no significant difference in the experienced problems with falling asleep or sleeping between 7th–8th grade and 9th–10th grade students and between students who live with both parents and one parent. Table 2 and Table 3 present a more comprehensive frequency of headaches, abdominal pain, and problems with falling asleep or sleeping in the last six months, a comparison between gender, grade, and family status.

A total of 497 (69.4%) boys indicated that they felt healthy, and 57 (8.0%) stated that they felt unhealthy, whereas 314 (47.4%) girls indicated that they felt healthy, and 91 (13.7%) stated that they felt unhealthy, *p* < 0.001. In addition, boys were statistically significantly more likely than girls to report that they felt happy (51.4% vs. 29.6%), *p* < 0.001. 380 (56.9%) Seventh to eighth grade students indicated that they felt healthy, and 261 (39.4%) stated that they felt happy. Meanwhile, 413 (60.1%) 9th–10th grade students indicated that they felt healthy, and 286 (41.7%) indicated that they felt happy. A total of 685 (60.0%) teenagers, or more than half, who live with both parents felt healthy, whereas those who live with only one parent—115 (52.3%). A total of 474 (41.6%) students who lived with both parents felt happy, and those who lived with only one parent—81 (37.2%). A more detailed comparison of student happiness and well-being between genders and grades is shown in Table 4.

### 3.3. Students’ Emotional, Behavioral Problems and Problematic Internet Use

The mean of the SDQ total difficulties score (TDS) of all surveyed students was 15.00 ± 5.53. Meanwhile, the mean internalizing score (IS) mean was 6.55 ± 3.41, and the mean externalizing score (IS) was 8.28 ± 3.26. The mean Internet Addiction Test (IAT) score of 7th–10th grade students was 40.86 ± 12.29. A total of 287 (20.8%) students, or a fifth, had total scores that ranged from 0 to 30 points and were considered to reflect a normal level of internet usage. Meanwhile, 784 (56.7%) students, or more than half, had a mild level of internet addiction (scores of 31 to 49). A total of 301 (21.8%) had a moderate level of addiction (scores of 50 to 79), and only 10 (0.7%) students scored from 80 to 100 points, indicating a severe dependence on the internet.

After performing the independent samples *t*-test and comparing the SDQ TDS between genders, we noticed that the average SDQ TDS for girls was statistically significantly higher than that of boys (16.37 ± 5.52 vs. 13.74 ± 5.33). Similar results were obtained when comparing SDQ IS (7.57 ± 3.55 vs. 5.59 ± 3.35), *p* < 0.001. Comparing the IAT, the average score of girls was also statistically significantly higher than boys (41.87 ± 12.24 vs. 39.96 ± 12.37). It was noticed that adolescents who lived with both parents had significantly lower IAT scores than those adolescents who lived with only one parent (40.71 ± 12.37 vs. 42.14 ± 12.37), *p* = 0.048. Similar results were obtained when comparing SDQ ES (8.34 ± 3.23 vs. 9.03 ± 3.35), *p* = 0.018. Statistically significant differences were not obtained when comparing SDQ TDS, SDQ IS, SDQ ES, and IAT between students in grades 7–8 and 9–10. Table 5 shows a more comprehensive comparison of students’ emotional and behavioral problems and problematic internet use by gender and grades.

### 3.4. Problematic Internet Use Association with Students’ Aches, Sleep, and Wellness

The ANOVA performed a comparison between the IAT score and students’ experienced problems with falling asleep or sleeping, and it showed that there was a statistically significant difference between the answer options. Post hoc pairwise comparisons showed that students with no sleep problems had lower IAT scores than those who experienced sleep problems less than once a week, 1–2 times a week, 3–5 times a week, or every night (*p* < 0.001). However, no statistically significant difference was found in the IAT score between students who experienced sleep difficulties 3–5 times a week and those who experiences difficulties every night (*p* = 0.091).

Similar results were obtained when the IAT score was compared with the frequency of headaches experienced by the students in the past 6 months (*p* < 0.001). Post hoc pairwise comparisons showed that the IAT results of students who hardly ever experienced headaches were lower than those who experienced headaches less than once a month, at least once a month, and at least once a week (*p* < 0.001). However, a statistically significant difference in the IAT score was not observed between students who suffered headaches less than once a month and those who suffered headaches at least once a month (*p* = 0.124).

Also, it was observed that those adolescents who hardly ever felt abdominal pain had a lower IAT score than those who suffered abdominal pain less than once a month, at least once a month, and at least once a week (*p* < 0.001). However, there was no significant difference in the IAT score between students experiencing abdominal pain less than once a month and those experiencing it at least once a month (*p* = 0.274).

Adolescents who reported feeling healthy during the questionnaire had statistically significantly lower IAT scores than those who felt only partially healthy or not healthy at all (*p* < 0.001). It was also observed that students who reported being happy had lower IAT scores than those who reported being only partially happy or not happy (*p* < 0.001). Complete information on problematic internet use and its association with students’ aches, sleep, and well-being is given in Table 6.

### 3.5. Problematic Internet Use Association with Strengths and Difficulties Questionnaire Score

A weak, statistically significant, positive correlation was found between students’ IAT scores and SDQ IS (r = 0.366, *p* < 0.001). The exact weak positive correlation was found between students’ IAT scores and SDQ ES (r = 0.385, *p* < 0.001). A significant, positive correlation was found between the students’ SDQ IS and ES estimates (r = 0.366, *p* < 0.001). In the group of girls, a moderate, statistically significant, positive correlation was found between IAT scores and SDQ ES (r = 0.402, *p* < 0.001). A weak positive correlation was found between IAT scores and SDQ IS (r = 0.323, *p* < 0.001). In the group of boys, moderately weak, statistically significant, positive correlations were found between IAT score and SDQ IS (r = 0.395, *p* < 0.001) and SDQ ES (r = 0.360, *p* < 0.001). However, statistically significant correlations were not determined between adolescent age and IAT scores, SDQ IS, and SDQ ES. A more explicit correlation matrix of the Internet Addiction Test score and explanatory variables is shown in Table 7.

The linear regression model met the required parameters when the dependent variable was adolescents’ IAT scores, and the regressors were SDQ IS, SDQ ES, and student age. A linear regression model could not be created when the regressors were gender, headache, abdominal pain, feeling of happiness, or health. A further linear regression (determination coefficient R^2^ > 0.221) shows the significant effect of the predictor SDQ IS (β standardized coefficient = 0.269, *p* < 0.001) and a more significant effect of the predictor SDQ ES (β standardized coefficient = 0.296, *p* < 0.001). Linear regression in the subgroup of girls (coefficient of determination R^2^ > 0.231) shows a significant predictor effect for SDQ IS (β standardized coefficient = 0.231, *p* < 0.001), whereas SDQ ES (β standardized coefficient = 0.339, *p* < 0.001) was a more significant regressor for girls’ IAT score. However, linear regression in the subgroup of boys (coefficient of determination R^2^ > 0.221) shows a significant predictor effect for SDQ ES (β standardized coefficient = 0.248, *p* < 0.001). Still, the impact of SDQ ES (β standardized coefficient = 0.306, *p* < 0.001) was more significant for boys’ IAT scores. Table 8 presents a complete regression analysis of the Internet Addiction Test score.

## 4. Discussion

It is important to note that children’s internet addiction is an emerging problem in Lithuania that causes health difficulties. Individuals who seem to be in a risk group for having social media addiction are teenagers, students, lonely people, and introverts. Even though adolescents do not agree that they prefer virtual communication over meeting in person, it was found that young people can spend the whole day communicating in social media and even forget to eat or do their homework [34]. One Lithuanian study revealed that about one-tenth (10.6%) of the boys and a bit less (7.7%) of the girls from 13 to 18 years have internet addiction [35]. Our study revealed that only one-fifth of the students who participated in our research do not have an addiction to the internet (IAT < 20). Still, only less than 1% of adolescents had a severe addiction to the internet. A similar study in Croatia, which has been analyzing adolescents’ internet addiction, has shown that a total of 24.4% of respondents (IAT  <  20) show no signs of addiction. According to the survey results, the majority of respondents (39%) were categorized as having a low level of addiction (20  ≤  IAT ≤ 39), and 32% showed a moderate level of addiction (40  ≤  IAT  ≤  69). Only 3.4% of respondents were classified as having a severe level of addiction (IAT ≥ 70) [36]. A study conducted in Germany in 2022 showed that 43.69% of 12–17-year-old teenagers used the internet in problematic ways [21]. In Portugal, a study found that adolescents exhibit varying degrees of internet addiction: normal users (35.9%), users with mild addiction (47.9%), and users with moderate addiction (16.2%) [37].

Our study revealed that slightly less than half of the boys indicated they had not experienced headaches and abdominal pain in the last 6 months. Meanwhile, girls expressed that they had these pains much more often. In addition, girls were significantly more likely to have problems falling asleep and staying asleep. The same situation was observed when assessing students’ happiness and health. Almost half of the boys said they felt happy, and even three-quarters said they were healthy. Meanwhile, only a third of the girls were happy, and half felt healthy. Gender differences are causing significant concern, and it is crucial to understand why teenage girls seem to experience much worse feelings than boys of the same age. It is possible that girls are more sensitive and more aware of their symptoms, or that boys are more denying or more tolerant with regard to their symptoms [38].

How are girls’ and boys’ internet addictions different? Mihara and colleagues also found that boys were more likely to spend their time on the internet playing games, whereas girls used it to create and maintain social connections [1]. However, both genders have been noted for risk factors for problematic internet use [39]. According to our study, we have observed that girls tend to have higher IAT scores than boys. After conducting a linear regression, we noticed that externalizing difficulties had a greater impact on the IAT estimate of girls, making it a more significant regressor. Conversely, internal difficulties were found to have a more significant impact on the IAT estimate of boys. Such research results suggest that girls who are more impulsive have more behavior problems and attention problems and may be more inclined to use the internet intensively. This may be explained by different coping strategies for stress or bad emotions. Studies on addiction have shown that both genders can be affected, with some research indicating higher rates of addiction among females [21,40,41]. It has been consistently observed in various studies that males exhibit significantly higher scores of internet addiction as compared to females [42,43]. Adolescents with insecure peer attachment can assume low social competence. According to some studies, such difficulties may occur more often in girls than in boys [44]. Difficulty developing or maintaining social contact can lead to loneliness [45]. Participating in online activities that involve simulated social interactions and friendships can provide a sense of belonging and distraction [46]. It is important to note that loneliness and difficulties with peers can serve as general risk factors for problematic internet use. In contrast, good relationships with peers and good emotion regulation appear protective for adolescents in this context. Enhancing the quality of real-life relationships can address problematic internet use from its root cause instead of just treating the symptoms. This suggests promoting real-life and positive peer relationships to prevent adolescents from problematic internet use, which might lead to better outcomes than restricting internet use alone [36].

In our study, we observed that adolescents who lived with only one parent had higher IAT scores, indicating that family has an important influence on internet addiction. This suggests that teenagers who are more likely to have emotional problems and are addicted to the internet are more likely to come from a family with divorced parents. In this age group, individuals face problems such as the inability to cope with negative emotions and emotional immaturity, and these factors may lead to a link between internet addiction and parental divorce. According to a study conducted in Lebanon that looked at the factors of internet addiction in adolescence, it is known that seven percent of internet-addicted teenagers live in dysfunctional or problematic families [47]. The fact that a dysfunctional family is one of the factors in the emergence of internet addiction is also proven by the fact that teenagers whose parents are married but live separately have the highest level of internet addiction [48]. Internet addiction among teenagers is more common than among adults. Teenagers need empathy and protection from family members during this transitional stage, and when the family does not meet these requirements, teenagers can compensate for the loss of love by using social networks [49]. Among psychosocial factors, socio-economic status also influences excessive use of the internet. A Shanghai-based study of 5135 students from the 7th to 12th grades found an association between internet use and socioeconomic status. Adolescents from high socioeconomic status families used the internet more than those from low socioeconomic status families [48]. However, another study found that it was teenagers whose family income was lower than expenses who had a higher risk of using the internet more [22].

According to our study, the students who reported feeling happy had lower IAT scores. Unhappiness and self-isolation have contributed to the internet taking over numerous areas of life. According to the research, one of the highest self-isolation and unhappiness factors was the COVID-19 pandemic lockdown, which led to digital technology overuse, especially among children and adolescents. As the most vulnerable members of society, they are in greater danger of losing self-control while using the internet [50]. Based on the research study “Children’s distance learning during the COVID-19 pandemic: threats and opportunities from an ecosystem perspective” carried out in Lithuania, children’s screen time has increased significantly over the last few years. During the spring quarantine period, primary school-aged children spent an average of 4 h per day in front of screens, and older children spent an average of 6 h. Screens were used not only for education but also for entertainment. Together with other academic and health professional communities, the World Health Organization recommends that screen time for leisure purposes between school-aged children should not exceed 2 h per day. Unfortunately, one in five pupils in Lithuania spends 5–6 h or more of their free time using digital devices [51]. Also, Lung and colleagues found that spending longer periods of time on the internet (>5 h) was associated with lower levels of subjective happiness [52]. These findings suggest that most adolescents often turn to the internet when they feel unhappy. Excessive use of the internet often makes teenagers feel more unhappy. It is like a closed circle, where one factor encourages the manifestation of another factor.

Our study found that girls had significantly higher scores in internalizing difficulties and total SDQ scale scores compared to boys. In the 2021 year analysis, it was observed that when comparing externalizing and internalizing problems, gender differences were significant: boys reported significantly more externalizing problems than girls, and girls reported more internalizing problems than boys [53]. In a study conducted in Holland, the comparison by gender revealed that especially in the age groups of 6–11 and 12–18, boys had less favorable SDQ scores than girls. Furthermore, we found that girls aged 12–18 had more emotional symptoms than boys. The study found that there were significant gender differences in hyperactivity-inattention, peer problems, prosocial behavior, and externalizing and total difficulties [54]. These gender differences were in line with results from previous research among European countries [55,56].

Our study observed a positive correlation between higher IAT scores and internalized and externalized difficulties. Additionally, students who experienced headaches or stomachaches and poor sleep exhibited higher IAT scores. Many systematic literature reviews show that internet overuse is linked to even worse psychosocial issues such as internet addiction, stress, aggression, hyperactivity and attention deficit, impulsivity, depression, and anxiety among adolescents. Collected data suggest that time spent online correlates with psychiatric health disorders [22,57]. It was claimed that frequent online activity is linked to several mental health problems, such as hyperactivity, antisocial behavior, and compulsive internet use [58]. This suggests a profound impact of internet addiction on the mental health of adolescents. Also, data from this study, which was carried out in Lithuania, Latvia, and Taiwan, indicate that children who were spending more time online are much more likely to be reported by their parents for conduct problems [58]. Emotional and behavioral difficulties, the use of IT devices, and problematic internet use overall are often associated with the emotional environment of the family in Lithuania [58]. Higher ratings of emotional and behavioral problems are exhibited by children who experience the emotional environment of a bad family, which leads to excessive internet usage. Furthermore, increased screen time, for both education and entertainment, has been linked to physical discomforts such as headaches and obesity, which means that internet addiction causes not only mental but also somatic problems [59]. In another Lithuanian study, it was revealed that many indicators of the level of psychological difficulties and specific personality traits are related to internet addiction. The strongest connection tends to be between internet addiction and neuroticism, whereas the weakest connection is between extraversion and internet addiction [60]. This was also confirmed by Blumer and Doring, who measured student participants by the NEO-Five Factor Inventory (NEO-FFI) [61]. Another trait that predicts children’s compulsive use of the internet in Lithuania, according to parents, is male gender, lower levels of prosocial behavior, and higher levels of emotional and behavioral problems while considering the family’s financial situation [62]. These results highlight the complex aspects of internet addiction in the Lithuanian younger generation, pointing to the necessity for specialized interventions that tackle the psychological, physical, and situational contributors to this problem. Luckily, it was declared that problematic internet usage can be treated. Researchers recommend that psychological and pharmacological interventions, which show significant impact, should be implemented early on, especially within elementary schools. This is due to the high prevalence of internet misuse among children and adolescents in Lithuania [63].

Concerning internet addiction, adolescents with internet addiction exhibited notably higher average scores across all SDQ subscales, except for the prosocial scale [64]. Furthermore, a notable positive correlation was observed between anticipation, excessive use, lack of control, neglect of social life, neglect of work, and salience domains of the Internet Addiction Test with conduct, hyperactivity, and peer problems. Only three IAT domains—excessive use, lack of control, and salience—demonstrated a significantly positive correlation with the emotional domain of the Strengths and Difficulties Questionnaire [65]. In another study, internet addiction was assessed using the Excessive Internet Use Scale (EIUS). The total difficulties score (TDS) of the SDQ has a possible scale from 0 to 40, originally divided into three categories: normal (0–15), borderline (16–19), and abnormal (20–40). Adolescents who reported borderline and abnormal levels of emotional and behavioral problems were found to be more inclined to report excessive internet use and internet addiction compared to those with no identified EBP issues [66]. Akdeniz’s team used “YIAT-SF” (Young’s Internet Addiction Test-Short Form) to evaluate internet addiction. Individuals who scored 36 or higher on the “YIAT-SF” were classified as belonging to the internet addiction group, whereas those scoring below 36 were designated as members of the non-internet addiction group. The internet addiction group demonstrated elevated scores in inattention/hyperactivity, emotional symptoms, conduct problems, peer relationship issues, and overall problems while exhibiting diminished scores in pro-social behavior when compared to the NIA group [67]. It is known that high screen time, prolonged periods of smartphones, and excessive internet use were positively associated with externalizing problems [68,69].

## 5. Conclusions

According to this study, internet addiction is becoming an increasingly concerning issue for adolescent mental and somatic health. Our study revealed that higher internet addiction was associated with both internalized and externalized difficulties. The study also revealed that adolescents with higher levels of internet addiction were more likely to be unhappy. They also rated their health as worse and frequently complained of sleep problems, abdominal pain, and headaches. It is probably important to mention that girls might be the group that has a higher probability of internet addiction and feels less happy and healthy. It is crucial to promptly identify and assist those who experience excessive internet usage that negatively affects their psychological, emotional, and physical health. Also, it is imperative that parents, children, and young people receive consistent, competent medical, psychological, and psychotherapeutic education about the severe health consequences of excessive and early media consumption. The results of this study can serve as initial steps toward developing preventive interventions.

## 6. Limitation

This study had several limitations. Our study was cross-sectional, allowing us to identify associations between various factors. However, we could not test causal relationships. It is possible that frequent internet addiction contributes to behavioral and emotional problems, happiness, or wellness, but it is also likely that adolescents with certain behavioral or emotional difficulties are more inclined to use the internet. Furthermore, previous research has indicated the need to recognize potential bidirectional relationships between adolescents’ behavior or emotional difficulties and internet use [70,71]. In our opinion, longitudinal studies could better evaluate the direction of the effect of internet addiction on adolescent mental health. Another limitation could be that we only included teenagers aged 12–16 in the study, which does not help us to understand the differences between the stages of adolescence. In addition, our understanding of adolescent internet use, emotional and physical health, happiness, and wellness was complicated by the fact that we did not include teachers or parents in the study who could provide their perspectives on adolescent-related issues. Another consideration is that we used non-standardized questionnaires to determine adolescents’ happiness or wellness.

## Figures and Tables

**Table 1:** The characteristics of the sample.

Variable	N	%
Gender		
Male	738	52.3
Female	674	47.7
Cities		
Vilnius	673	47.7
Klaipėda	408	28.9
Molėtai	331	23.4
Student’s grade		
7	372	26.8
8	315	22.7
9	361	26.0
10	339	24.4
Family status		
Living with both parents	1186	83.7
Living with one parent	227	16.3

**Table 2:** A comparison of headaches and abdominal pain between gender, grade, and family status.

**During the Past Six Months, Have You Experienced Distracting Headaches?**									
**Factors**	**Hardly Ever**		**Less Than Once a Month**		**At Least Once a Month**		**At Least Once a Week**		***p***
	***n***	**%**	***n***	**%**	***n***	**%**	***n***	**%**	
Gender									
Male	309	42.0	164	22.3	169	23.0	94	12.8	<0.001
Female	109	16.3	123	18.4	214	32.0	223	33.3	
Student’s grade									
7th–8th grades	227	33.3	134	19.7	175	25.7	145	21.3	0.053
9th–10th grades	186	26.6	146	20.9	200	28.6	168	24.0	
Family status									
Living with both parents	358	30.8	233	20.0	314	27.0	258	22.2	0.373
Living with one parent	57	25.3	51	22.7	61	27.1	56	24.9	
**During the Past Six Months, Have You Experienced Recurring Abdominal Pain?**									
Gender									
Male	323	44.1	228	31.1	133	18.1	49	6.7	<0.001
Female	112	16.8	109	16.3	294	44.0	153	22.9	
Student’s grade									
7th–8th grades	199	29.3	177	26.1	209	30.8	94	13.8	0.283
9th–10th grades	229	32.8	154	22.1	213	30.5	102	14.6	
Family status									
Living with both parents	368	31.7	284	24.5	344	29.7	164	14.1	0.256
Living with one parent	64	28.6	46	20.5	79	35.3	35	15.6	

**Table 3:** Experienced problems with falling asleep or sleeping between gender, grades, family status.

During the Past Six Months, Have You Experienced Problems with Falling Asleep or Sleeping?											
Factors	Never		<1 a Week		1–2 a Week		3–5 a Week		Every Night		*p*
	*n*	%	*n*	%	*n*	%	*n*	%	*n*	%	
Gender											
Male	266	36.2	153	20.8	160	21.8	94	12.8	62	8.4	<0.001
Female	128	19.1	150	22.4	166	24.8	116	17.3	110	16.4	
Student’s grade											
7th–8th grades	210	30.8	152	22.3	155	22.8	84	12.3	80	11.7	0.062
9th–10th grades	177	25.3	145	20.7	164	23.4	124	17.7	90	12.9	
Family status											
Living with both parents	335	28.8	248	21.3	263	22.6	179	15.4	139	11.9	0.528
Living with one parent	57	25.4	51	22.8	57	25.4	28	12.5	31	13.8	

**Table 4:** Comparison of student happiness and well-being between gender, grades, and family status.

Are You Feeling Healthy?								Are You Feeling Happy?						
Factors	Yes		Partially		No		*p*	Yes		Partially		No		*p*
	*n*	%	*n*	%	*n*	%		*n*	%	*n*	%	*n*	%	
Gender														
Male	497	69.4	162	22.6	57	8.0	<0.001	367	51.4	228	31.9	119	16.7	<0.001
Female	314	47.4	257	38.8	91	13.7		195	29.6	258	39.2	110	31.3	
Student’s grade														
7th–8th grades	380	56.9	207	31.0	81	12.1	0.238	261	39.4	223	33.6	179	27.0	0.054
9th–10th grades	413	60.1	209	30.4	65	9.5		286	41.7	259	37.8	141	20.6	
Family status														
Living with both parents	685	60.0	339	29.7	118	10.3	0.096	474	41.6	400	35.1	265	23.3	0.404
Living with one parent	115	52.3	76	34.5	29	13.2		81	37.2	79	36.2	58	26.6	

**Table 5:** Students’ emotional and behavioral problems and problematic internet use are compared between gender, grades, and family status.

Factors	IAT		IS		ES		TDS	
	Mean	SD	Mean	SD	Mean	SD	Mean	SD
Gender								
Male	39.96	12.37	5.59	3.35	8.15	3.13	13.74	5.33
Female	41.87	12.24	7.57	3.55	8.56	3.37	16.37	5.52
*p*	0.004		<0.001		0.053		<0.001	
Class of students								
7th–8th grade	41.26	12.54	6.64	3.72	8.62	3.22	15.25	5.66
9th–10th grade	40.50	12.03	6.45	3.46	8.28	3.31	14.76	5.49
*p*	0.325		0.325		0.084		0.100	
Family status								
Living with both parents	40.71	12.29	6.49	3.59	8.34	3.23	14.84	5.53
Living with one parent	42.14	12.47	6.84	3.53	9.03	3.35	15.83	5.56
*p*	0.048		0.184		0.018		0.011	

IAT: Internet Addiction Test. IS: Internalizing score (SDQ). ES: Externalizing score (SDQ). TDS: Total difficulties score (SDQ). SD: Standard deviation.

**Table 6:** Problematic internet use is associated with students’ aches, sleep, and wellness.

**Have You Experienced Problems with Falling Asleep or Sleeping in the Past Six Months?**												
	**Never**		**<1 Time a Week**		**1–2 Times a Week**		**3–5 Times a Week**		**Every Night**		***p***	**Post Hoc Tukey‘s Test**
	**Mean**	**SD**	**Mean**	**SD**	**Mean**	**SD**	**Mean**	**SD**	**Mean**	**SD**		
IAT	36.50	11.01	39.81	11.14	42.35	12.61	43.57	11.22	46.88	14.33	<0.001	Never < 1 time a week < 1–2 times a week < 3–5 times a week * Every night
**During the Past Six Months, Have You Experienced Distracting Headaches?**												
	**Hardly Ever**		**Less Than Once a Month**		**At Least Once a Month**		**At Least Once a Week**					
	**Mean**	**SD**	**Mean**	**SD**	**Mean**	**SD**	**Mean**	**SD**				
IAT	39.81	11.14	42.35	12.61	43.57	11.22	46.88	14.33			<0.001	Hardly ever < Less frequently * At least once a month < At least once a week
**During the Past Six Months, Have You Experienced Recurring Abdominal Pain?**												
	**Hardly Ever**		**Less Than Once a Month**		**At Least Once a Month**		**At Least Once a Week**					
	**Mean**	**SD**	**Mean**	**SD**	**Mean**	**SD**	**Mean**	**SD**				
IAT	36.79	11.42	41.22	11.36	41.65	12.08	44.95	13.18			<0.001	Hardly ever < Less frequently * At least once a month < At least once a week
**Are You Feeling Healthy?**												
	**Yes**		**Partially**		**No**							
	**Mean**	**SD**	**Mean**	**SD**	**Mean**	**SD**						
IAT	38.26	11.52	43.91	11.64	46.47	11.56					<0.001	Yes < Partially; No
**Are You Feeling Happy?**												
	**Yes**		**Partially**		**No**							
	**Mean**	**SD**	**Mean**	**SD**	**Mean**	**SD**						
IAT	36.84	11.17	42.20	11.79	45.72	11.84					<0.001	Yes < Partially < No

* Significant differences *p* > 0.05 IAT: Internet Addiction Test. SD: Standard deviation.

**Table 7:** Correlation matrix of Internet Addiction Test score and explanatory variables.

	Total (*n* = 1384)				Female (*n* = 643)				Male (*n* = 715)			
	IAT	IS	ES	Age	IAT	IS	ES	Age	IAT	IS	ES	Age
IAT	1.000	0.366 *	0.385 *	−0.034	1.000	0.323 *	0.402 *	−0.011	1.000	0.395 *	0.360 *	−0.049
IS		1.000	0.329 *	0.004		1.000	0.274 *	0.047		1.000	0.358 *	−0.009
ES			1.000	−0.035			1.000	0.012			1.000	−0.072
Age				1.000				1.000				1.000

IAT: Internet Addiction Test. IS: Internalizing score (SDQ). ES: Externalizing score (SDQ). * *p* < 0.001.

**Table 8:** Regression analysis of Internet Addiction Test score.

	Total (*n* = 1384)			Girls (*n* = 643)			Boys (*n* = 715)		
	B	SE_b_	β	B	SE_b_	β	B	SE_b_	β
Intercept	29.333	3.705		28.942	5.295		29.952	5.193	
IS	0.922	0.088	0.269 *	0.800	0.126	0.231 *	1.120	0.132	0.306 *
ES	1.107	0.096	0.296 *	1.221	0.132	0.339 *	0.969	0.140	0.248 *
Age	−0.258	0.248	−0.025	−0.267	0.359	−0.026	−0.284	0.344	−0.028

IS: Internalizing score (SDQ). ES: Externalizing score (SDQ). * *p* < 0.001, B = unstandardized regression coefficient, SEB = standardized error of the coefficient, and β standardized coefficient.

## Data Availability

The raw data supporting the conclusions of this article will be made available by the authors, without undue reservation.
